# The Determination of Protonation Constants of Peptidomimetic Cyclophanes in Binary Methanol-Water Mixtures

**DOI:** 10.1155/2016/1721069

**Published:** 2016-07-18

**Authors:** Piotr Seliger, Danuta Tomczyk, Grzegorz Andrijewski, Ewa Tomal

**Affiliations:** ^1^Department of Inorganic and Analytical Chemistry, University of Lodz, Tamka 12, 91-403 Lodz, Poland; ^2^Department of Biochemistry and Genetics, Institute of Biology, Jan Kochanowski University in Kielce, Swietokrzyska 15 A, 25-406 Kielce, Poland

## Abstract

The protonation constants of new group of peptidomimetic cyclophanes with valine or phenylalanine moieties incorporated into the macrocyclic skeleton as well as their linear analogues were determined by potentiometric measurements in solutions of methanol-water mixtures at 25°C and constant ionic strength. The influence of cavity size, location of protonation sites, and attached substituents of the macrocyclic ligands on the protonation constants were discussed on the basis of potentiometric measurement as well as H^1^-NMR results.

## 1. Introduction

The acidity constants of organic reagent play an important role in many analytical procedures such as solvent extraction, complex formation, ion transport, and acid-base titration. The influence of acid-base properties affects the toxicity of the compounds [1] and pharmaceuticals properties (adjusting the dosage form to provide optimum bioavailability) [2]. Since most drugs are hardly soluble in water the pKa values are often determined in mixtures of water and an organic solvent, mainly alcohol, to get suitable solubility [3, 4]. Methanol is widely accepted as a cosolvent and its effect on pKa has been investigated extensively [5, 6].

In recent years the interest of peptidomimetic compounds arouse in different areas of research. This class of compounds found applications as the receptors in molecular recognition of cations and anions [7], as well as in other areas of supramolecular chemistry, for example, in self-assembling of nanotubes and rosettes [8]. Another very interesting aspect noticed for this class of compounds is their important biomedical activities. Some of the molecules containing peptides or peptide-related fragments within their structure show antiviral activity [9] as well as antibacterial properties [10]. The part of compounds presented in this paper is known to suppress hepatitis C virus RNA replication [11] and regulate maturation and function of monocyte-derived dendritic cells [12] as well as new drugs in chemotherapy of breast cancer [13].

In this context finding out acid-base equilibria of this class of compounds seemed to be very crucial for their further possible application and environmental conditions necessary for proper activity.

## 2. Experimental Section

### 2.1. Materials

All investigated linear and cyclophane ligands were synthesized according to the procedures previously described [14–16] as well as the pyridinophane derivatives [17]. The tetraethylammonium perchlorate was commercial sample (Fluka) of electrochemical grade. Tetraethylammonium chloride (Aldrich), perchloric acid 72% (Fluka), 2,6-dinitrophenol 97% (Aldrich), potassium hydrogen phthalate 99.95% (Aldrich), oxalic acid and ammonium oxalate monohydrate ≥99% (POCh Gliwice), succinic acid 98% (Lancaster), and lithium hydrogen succinate (Aldrich) were used without purification. Methanol 99.8 HPLC gradient grade (JT Baker) and deuterated methanol (Aldrich) were commercial samples. The water used in measurements was triple distilled in glass.

### 2.2. Methods

^*1*^ *H NMR spectra* were recorded on a Bruker 200 MHz spectrometer using solutions in CD_3_OD with TMS as internal reference.

*Potentiometric* measurements were carried out with the use of microburette controlled by computer with the following measurement system:

(1) $$\begin{array}{c} \begin{array}{c} Ag\;|\;AgCl\;|\;\underset{in\;\;x_{0.1MeOH\;\;or\;\;0.95MeOH}}{{\left(C_{2}H_{5}\right)}_{4}NCl}||\underset{in\;\;x_{0.1MeOH\;\;or\;\;0.95MeOH}}{C_{H}+C_{L}+C_{{\left(C_{2}H_{5}\right)}_{4}{NClO}_{4}}}||\;\;glass\;\;electrode \end{array} \end{array}$$

The main problem during pH-metric titration in mixed solvent solutions with glass electrode, as the working electrode, is its calibration. There are several procedures reported in the literature based on different methods used to gain the full characteristic of the electrode. We have used two methods. The first one is based on prepared buffer solutions [18–20] (phthalate and oxalate buffers for *x*_0.1MeOH_ and oxalate and hydrogen succinate buffers for *x*_0.95MeOH_) and the second one is based on titration of buffer solution described by Wróbel and others described elsewhere [21] (titration of 1 · 10^−3^ mol·dm^−3^ solution of tetra-n-butylammonium 2,6-dinitrophenolate by the mixture of 1 · 10^−3^ mol·dm^−2^ of 2,6-dinitrophenol and 1 · 10^−3^ mol·dm^−3^ solution of tetra-n-butylammonium 2,6-dinitrophenolate). In both cases we obtained similar calibration parameters for the glass electrode used during experiment.

In a typical measurement carried out at 25°C 10 ml of 1.00 × 10^−3^ mol/dm^3^ ligand solution in the constant ionic strength was titrated with perchloric acid. Tetraethylammonium perchlorate salt was used as supporting electrolyte in concentration of 5.00 × 10^−2^ mol/dm^3^. All measurements were carried out with the use of Cerko Lab System instrument equipped with microburette controlled by computer.

### 2.3. Method of Protonation Constant Calculation

All calculations were done by our computation program based on the following assumption.

In each point of titration curve of weak base by strong acid the following equation must be satisfied:

(2) $$\begin{array}{c} \frac{a\cdot c-\left[H^{+}\right]+K_{w}/\left[H^{+}\right]}{c}=\frac{\sum_{i=1}^{n}i\prod K_{i}{\left[H^{+}\right]}^{i}}{1+\sum_{i=1}^{n}\prod K_{i}{\left[H^{+}\right]}^{i}}, \end{array}$$

where *c* is the overall base concentration, *K*_*i*_ is the successive protonation constants, *a* is the titration fraction, *K*_*s*_ is the ionic product of solvent, [H^+^] is the equilibrium concentration of hydrogen ions, and *n* is the number of protons which can be attached to the base molecule.

If the base concentration, the titration fraction, and the values of protonation constants are known this equation could serve us for calculation of pH in arbitrary point of titration curve. The easiest way to gain this aim is the application of the quick convergent Newton-Raphson method. The iterative equation in its modified form [22] is as follows:

(3) $$\begin{array}{c} {\left[\;H^{+}\;\right]}_{j}=\frac{{\left[H^{+}\right]}_{j-1}}{\exp\left(F{\left[H^{+}\right]}_{j-1}/F^{\prime }{\left[H^{+}\right]}_{j-1}\right)}, \end{array}$$

where

(4) FH+=a·c−H++Kw/H+c−∑i=1ni∏KiH+i1+∑i=1n∏KiH+iF′H+=∂FH+∂ln⁡H+.

Equation (2) could be also used for calculation of unknown protonation constants. The procedure to achieve this goal consists of the following steps:

(1) The initial set of protonation constants is input into the software program.
(2) For each of the experimental points of titration curve the pH value is calculated on the basis of the current set of protonation constants. In this way the calculated titration curve is obtained.
(3) Then the sum of square deviation between the calculated and experimental curve is computed:
  (5) $$\begin{array}{c} SD=\overset{n}{\underset{j=1}{\sum }}\left(\;pH{\left[\;j\;\right]}_{calc}-pH{\left[\;j\;\right]}_{exp}\;\right). \end{array}$$
(4) To the set of protonation constants the corrections are introduced in such a way to lower the SD value. Then steps (2)–(4) are repeated. In this point we used the algorithm proposed by Motekajtis and Martell [23].

In the computation, the applied value of the ionic product solvent was determined experimentally from the titration curve of strong base with the strong acid in each of the solvents mixtures.

**Scheme 1:**
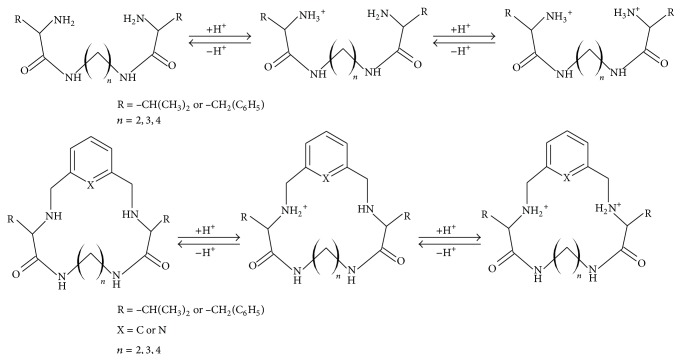
The schematic presentation of protonation of the investigated ligands.

The computation of protonation constants is based on the fitting of the experimental curve with the theoretical one. In the case of our study we have tried different models of protonation equilibria but the fitting parameters were acceptable only for the presented models (Scheme 1). Assumption of the other models gave no reliable results at all.

## 3. Results and Discussion

**Figure 1:**
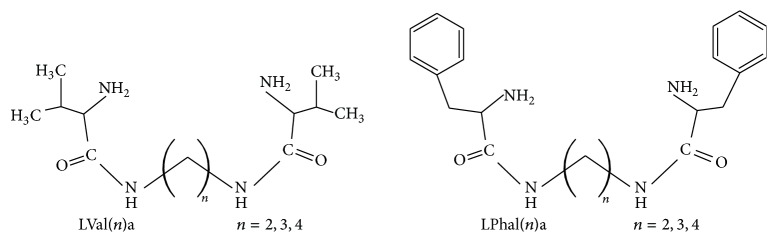
Structure of investigated linear ligands.

Structures of investigated linear ligands are presented in Figure 1. The values of successive protonation constants for linear ligands determined in the mixed methanol-water solution (*x*_0,95MeOH_) are presented in Table 1.

The proposed model of protonation of these derivatives is presented on Scheme 1.

As can be noticed, the values of successive protonation constants for each presented in Table 1 ligands are close to each other. It is the effect of the length of separation chain between protonation sites. This observation is in accordance with previously published data for diamine linear ligands with similar length of –(CH_2_)_*n*_– spacer [24, 25].

Introduction of two amide groups into ligand molecule significantly reduce value of successive protonation constants for all ligands. Amide group contains oxygen atom, which is a very strong acceptor of hydrogen bond and nitrogen atom which can be a donor during the forming of such bond. The presence of these groups in molecule separated from each other by the chain of suitable length can lead to intramolecular hydrogen bond. But existence of intermolecular hydrogen bond depends not only on a suitable length of separation chain, but also on properties of the solvent and, as it is in this case, on the presence of hydrophobic substituents within the ligand structure. The suitable length of separation chain provides a free rotation of amide groups and possibility of formation of hydrogen bond between them. This possibility was studied in literature [26] and as it occurs in the case of investigated ligands the length of the methylene spacer allows such behavior. At the same time, the properties of methanol-water solution more likely will lead to hydrogen bond within ligand structures and then to intermolecular one. So, the presences of hydrophobic group in ligands molecules will be a main factor which can lead to forming of this kind of bonding in case of investigated ligands. Each ligand has got two hydrophobic groups in the skeleton, isopropyl, and benzyl in LVal(*n*)a and LPhal(*n*)a, respectively. These groups would be responsible for changing properties of donor atoms as well as lipophilic/lipophobic equilibrium [16, 27]. Hydrophobic groups will arrange a ligand skeleton in the way to reduce their interaction with polar solvent (broken conformation) and cause formation of intramolecular hydrogen bond. Hence, protonated ligands to avoid repulsion need to break the intramolecular hydrogen bond and change conformation from broken one to linear. This can be an explanation of slight reduction of basic properties of studied ligands.

**Figure 2:**
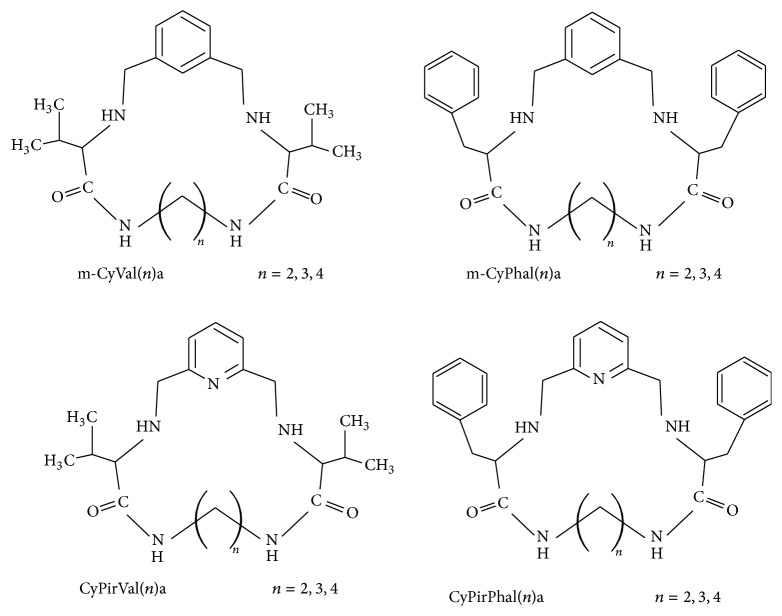
Structures of investigated cyclic ligands.

The second group of investigated compounds was macrocyclic ligands. Each structure of these cyclic ligands consists of two amide groups, two secondary amine groups, and endocyclic pyridine or benzene ring. All these moieties are part of intramolecular cavity of the investigated cyclic ligands. Furthermore, each of the macrocyclic compounds, similarly as linear ligands, possesses hydrophobic substituents (isopropyl or benzyl). Structures of investigated cyclic ligands are presented in Figure 2.

Values of successive protonation constants for cyclic ligands determined in methanol-water mixtures (*x*_0,1MeOH_ and *x*_0,95MeOH_) are presented in Table 2.

Values of successive protonation constants show significant decrease of basic properties of studied cyclic ligands as compared to their linear analogues. This is an effect of the presence of diverse donor groups in macrocyclic cavity and their induction effect, as well as less flexible conformation of the ligands and properties of the solvent. The influence of amide group and pyridine ring introduced into cavity of macrocyclic molecules was already published [28–35]. Appearance of amide group leads to reduction of protonation constants in comparison with unsubstituted analogues. The same effect is observed with pyridine ring in cavity of cyclic ligands and their unsubstituted analogues. From the values presented in Table 2 it can be seen that benzene ring incorporated into endocyclic part of macrocyclic ligands lowers slightly less than the value of protonation constants and then the pyridine ring. The two determined successive protonation constants in all of the studied ligands correspond to nitrogen's atoms from amine groups. Data from literature confirm that pyridine do not undergo protonation [34, 35]. As it was discussed above, in the cases of linear ligands, also the appearance of intermolecular bonding in cyclic ligands is possible. In this case, it depends on free rotation of amide group in cavity. Such possibility is connected with the flexibility (the size) of macrocyclic ring. As it was mentioned before, the presence of hydrophobic substituents as well as properties of solution is very important.

**Figure 3:**
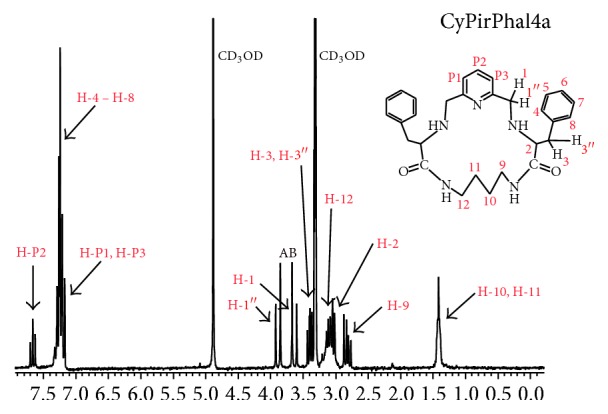
^1^H NMR spectrum of CyPirPhal4a in CD_3_OD.

**Figure 4:**
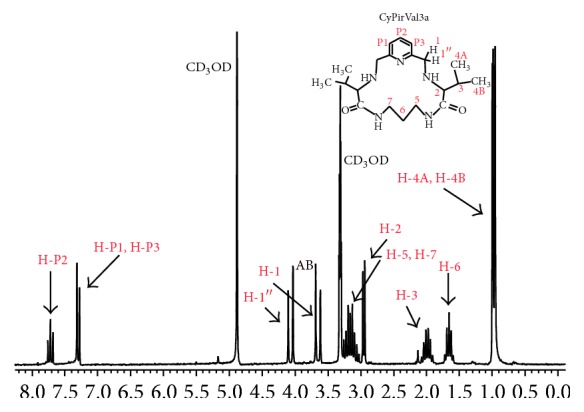
^1^H NMR spectrum of CyPirVal3a in CD_3_OD.

^1^H NMR spectra of CyPirVal(*n*)a and CyPirPhal(*n*)a were used for determination of ligands conformation. In all studied ligands hydrogen atoms in *α* position to pyridine ring (H-1 and H-1′′) can be seen as a very characteristic system of two doublets AB (with high coupling constants of about 14 Hz) in Figures 3 and 4.

It can lead to the assumption that structure of ligands is bent (boat conformation) on carbon atoms which bonds H-1 and H-1′′ protons. In the literature [16] signals attributed to hydrogen atoms located in *α* position according to nitrogen atom of amide group were used as an indicator of intramolecular hydrogen bond. Signals of those protons are split in ^1^H NMR spectrum of CyPirPhal(*n*)a ligands (protons: H-9 and H-12 in CyPirPhal4a, Figure 3). This can lead to assumption that one of carbonyl group is situated outside of macrocyclic cavity and the other is inside of it. In that case there is a possibility of appearance of intermolecular hydrogen bond. In spectrum of CyPirVal(*n*)a, signals, which are attributed to these protons, can be observed as multiplets (protons H-5 and H-7 in CyPirVal3a Figure 4). Probability of appearance of intermolecular bond is very weak.

On that base, it can be found that benzyl substituents in CyPirPhal(*n*)a increases hydrophobicity of those ligands. This effect is expressed in the values of successive protonation constants of both types of cyclic ligands. As the structure of CyPirPhal(*n*)a is more closed, the observed values are lower than those for CyPirVal(*n*)a in both solutions. What is expected, the discussed effect is even stronger in more polar medium *x*_0,1MeOH_. The similar effect can be seen for m-CyVal(*n*)a and m-CyPhal(*n*)a.

## 4. Conclusions

The protonation of the investigated compounds is realized through the amino groups present in the linear as well as the cyclic derivatives. The calculated values of successive protonation constants for all investigated ligands showed weak basic properties of these groups (from 6.18 to 8.44 for the first protonation constant). This is an effect of incorporation into ligands skeleton, the amide groups in the cases of linear derivatives, and also pyridine and benzene ring in cyclic ones. The highest values of the protonation constants are observed for linear ligands in methanol-water mixture *x*_0,95MeOH_.

The change of the reaction medium from *x*_0,1MeOH_ to *x*_0,95MeOH_ leads to the increase of the protonation constant of about 1 logarithmic unit.

In both types of ligands, the presence of hydrophobic substituents and polar properties of the mixed solvent solutions lead to intramolecular hydrogen bonds, which are more likely observed in derivatives with benzyl substituents, the lowest pKa values.

In the case of derivatives with pyridine moiety incorporated in the macrocyclic skeleton, there was no protonation of this fragment observed.

The calibration methods of the glass electrode used during the pH metric determination of protonation constants for the investigated compounds (IUPAC buffer solutions or titration of buffer solution described by Wróbel et al. [21]) gave reproducible parameters for the working electrode. Such results confirm the possibility of using one of the proposed methods during the determination of calibration parameters for the glass electrode in such mixed solvents environment.

## Figures and Tables

**Table 1:** Logarithm values of successive protonation constants of LVal(*n*)a and LPhal(*n*)a in methanol-water solution *x*_0,95MeOH_.

Linear ligands	log⁡*K*_*P*1_	log⁡*K*_*P*2_
LVal4a	8,44 ± 0,02	8,17 ± 0,02
LVal3a	8,17 ± 0,03	7,80 ± 0,03
LVal2a	8,14 ± 0,05	8,15 ± 0,03
LPhal4a	8,35 ± 0,02	7,86 ± 0,02
LPhal3a	8,11 ± 0,02	8,00 ± 0,01
LPhal2a	8,04 ± 0,04	7,82 ± 0,03

**Table 2:** Logarithm values of successive protonation constants CyPirVal(*n*)a, CyPirPhal(*n*)a, m-CyVal(*n*)a, and m-CyPhal(*n*)a in methanol-water solution *x*_0,1MeOH_ and *x*_0,95MeOH_.

Cyclic ligands	*x*_0,1MeOH_		*x*_0,95MeOH_	
	log⁡*K*_*P*1_	log⁡*K*_*P*2_	log⁡*K*_*P*1_	log⁡*K*_*P*2_
CyPirVal4a	6,56 ± 0,02	5,11 ± 0,02	7,38 ± 0,01	6,29 ± 0,01
CyPirVal3a	6,56 ± 0,02	5,40 ± 0,02	7,59 ± 0,01	6,50 ± 0,01
CyPirVal2a	6,37 ± 0,02	5,30 ± 0,02	7,57 ± 0,03	6,37 ± 0,03
CyPirPhal4a	6,18 ± 0,04	4,54 ± 0,05	7,31 ± 0,01	5,98 ± 0,01
CyPirPhal3a	6,24 ± 0,02	4,88 ± 0,03	7,23 ± 0,01	6,02 ± 0,01
CyPirPhal2a	6,15 ± 0,03	4,60 ± 0,03	7,13 ± 0,02	6,04 ± 0,02
m-CyVal4a	6,95 ± 0,03	5,53 ± 0,03	7,63 ± 0,02	6,18 ± 0,02
m-CyVal3a	6,91 ± 0,04	5,43 ± 0,04	7,99 ± 0,03	6,02 ± 0,04
m-CyVal2a	6,81 ± 0,03	5,57 ± 0,03	7,93 ± 0,04	5,73 ± 0,06
m-CyPhal4a	—^a^	—^a^	7,57 ± 0,03	5,67 ± 0,04
m-CyPhal3a	—^a^	—^a^	7,23 ± 0,05	5,97 ± 0,06
m-CyPhal2a	—^a^	—^a^	6,90 ± 0,06	6,05 ± 0,07

—^a^ denotes that the lack of the protonation constants for m-CyPhal(*n*)a derivative is connected with the insufficient solubility of the ligand in the medium.
